# Preliminary analysis of salivary microbiota in catathrenia (nocturnal groaning) using machine learning algorithms

**DOI:** 10.1080/20002297.2025.2489613

**Published:** 2025-04-16

**Authors:** Min Yu, Yujia Lu, Wanxin Zhang, Xu Gong, Zeliang Hao, Liyue Xu, Yongfei Wen, Xiaosong Dong, Fang Han, Xuemei Gao

**Affiliations:** a Department of Orthodontics, Peking University School and Hospital of Stomatology, Beijing, P.R. China; b Center for Oral Therapy of Sleep Apnea, Peking University Hospital of Stomatology, Beijing, P.R. China; c National Center for Stomatology, Beijing, P.R. China; d Department of Stomatology, Xuanwu Hospital, Capital Medical University, Beijing, P.R. China; e Sleep Division, Peking University People’s Hospital, Beijing, P.R. China

**Keywords:** Sleep-disordered breathing, biomarker, microbiota, upper airway, Nocturnal groaning

## Abstract

**Objectives:**

The present study aimed to characterize the salivary microbiota in patients with catathrenia and to longitudinally validate potential biomarkers after treatment with mandibular advancement devices (MAD).

**Materials and methods:**

Twenty-two patients with catathrenia (12 M/10 F, median age 28 y) and 22 age-matched control volunteers (8 M/14 F, median age 30 y) were included in the cross-sectional study. Video/audio polysomnography was conducted for diagnosis. All patients received treatment with custom-fit MAD and were followed for one month. Ten patients (6 M/4 F) underwent post-treatment PSG. Salivary samples were collected, and microbial characteristics were analyzed using 16S rRNA gene sequencing. The 10-fold cross-validated XGBoost and nested Random Forest Classifier machine learning algorithms were utilized to identify potential biomarkers.

**Results:**

In the cross-sectional study, patients with catathrenia had lower α-diversity represented by Chao 1, Faith’s phylogenetic diversity (pd), and observed species. Beta-diversity based on the Bray-Curtis dissimilarities revealed a significant inter-group separation (*p* = 0.001). The inter-group microbiota distribution was significantly different on the phylum and family levels. The treatment of MAD did not alter salivary microbiota distribution significantly. Among the most important genera in catathrenia and control classification identified by machine learning algorithms, four genera, *Alloprevotella, Peptostreptococcaceae_XI_G1, Actinomyces* and *Rothia*, changed significantly with MAD treatment. Correlation analysis revealed that *Alloprevotella* was negatively related to the severity of catathrenia (r^2^= −0.63, *p* < 0.001).

**Conclusions:**

High-throughput sequencing revealed that the salivary microbiota composition was significantly altered in patients with catathrenia. Some characteristic genera (*Alloprevotella, Peptostreptococcaceae_XI_G1, Actinomyces,* and *Rothia*) could be potential biomarkers sensitive to treatment. Future studies are needed to confirm and determine the mechanisms underlying these findings.

## Key messages

- Catathrenia (nocturnal groaning) is a rare, under-diagnosed sleep-related breathing disorder. The characteristics of salivary microbiota have emerged as a potential biomarker for sleeping disorders.
- This study utilized the 16S rRNA sequencing technique to characterize the salivary microbiota in patients with catathrenia, identified marker bacteria using machine learning algorithms, and validated the genera longitudinally after treatment with mandibular advancement devices (MAD).
- The results revealed clear differences in the diversity and composition of salivary microbiota between catathrenia and non-snoring control. Four genera, including *Alloprevotella*, *Peptostreptococcaceae_XI_G1*, *Actinomyces* and *Rothia*, changed significantly with MAD treatment and were correlated with groaning events.

## Introduction

Catathrenia, also known as sleep-related nocturnal groaning, is a rare sleep-related breathing disorder (SRBD) [1,2], characterized by recurring episodes of groaning events during sleep. Groaning events are defined by a deep inspiration, followed by a prolonged expiration, during which a monotonous groaning sound is made [3], which would make patients with catathrenia suffer from social embarrassment [4]. Furthermore, some patients complained about unrefreshing sleep and fatigue during the daytime [4], which might be related to the sleep fragmentation caused by the close association between arousal and groaning events [5,6]. Treatment options, such as positive airway pressure (PAP) and mandibular advancement devices (MAD), could relieve symptoms [7–9].

The diagnosis of catathrenia requires full-night video/audio polysomnography (PSG), which is time-consuming and expensive. The interpretation of groaning events should comprehend multiple signals, including airflow, sound, and oxygen saturation, because groanings could manifest as central apneas on the PSG [10], resulting in an underdiagnosis of potential patients with catathrenia [4]. One of the factors that may determine the diagnosis and severity of the disease is the human microbiome, which is known to play a major role in the modulation of systematic diseases [11]. Thus, conducting research on catathrenia at the molecular biology and microbiology levels could help establish meaningful biomarkers as diagnostic tools in the future.

Microbiota plays an important role in disease progression. Previous studies have documented microbiota dysbiosis related to diabetes, inflammatory bowel diseases, insomnia, autism spectrum disorders, and SRBD [12–15]. Adult and pediatric patients with obstructive sleep apnea (OSA) were found to have dysbiosis in salivary and gut microbiota, and effective treatment could reverse the imbalance [16–20]. Evidence from animal models also showed that intermittent hypoxia could affect gut microbiota [21,22]. While previous studies have primarily concentrated on the gut microbiota composition, the oral cavity is home to over 1,000 bacterial species, making it the second largest and most diverse microbial community in the human body, after the gut [23]. Therefore, salivary microbiota, painless and non-invasive to collect, has emerged as a potential biomarker for sleeping disorders. Machine learning methods are novel techniques to integrate omics datasets to offer a more comprehensive view of complex diseases and to enable the discovery of new biomarkers [24]. However, there is a lack of studies on the salivary microbiota characteristics in patients with catathrenia.

Thus, this exploratory study aimed to (1) characterize the salivary microbiota between catathrenia and age-matched control groups by 16S rRNA gene sequencing; (2) integrate machine-learning algorithms to identify potential biomarkers; and (3) validate potential biomarkers longitudinally with the treatment of MAD. The results demonstrated clear differences in diversity and composition of the bacterial species present in the saliva between catathrenia and control, and four characteristic genera were identified and validated longitudinally.

## Materials and methods

**Figure 1.**
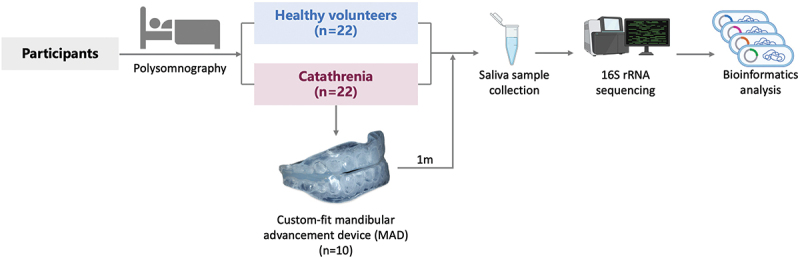
Overview of the study design.

The study design is illustrated in Figure 1. Firstly, a cross-sectional study was conducted to compare the salivary microbiota characteristics between patients with catathrenia and age-matched non-snoring control. Secondly, a longitudinal study was conducted to analyze the changes in salivary microbiota before and after treatment, and validate characteristic genera.

## Study population

This study was conducted at the Department of Orthodontics, Peking University Hospital of Stomatology. This study was approved by the Institutional Review Board of Peking University School of Stomatology (No. PKUSSIRB 201631128), and registered at the Chinese Clinical Trial Registry (No. ChiCTR-COC-17013239). The present study adhered to the declarations of Helsinki. Written informed consent was obtained from all the participants.

The sample size calculation was based on the Micropower package, a simulation-based method for permutational multivariate analysis of variance (PERMANOVA) based β diversity comparisons [25]. The Human Microbiome Project (HMP) dataset was used for distance matrix simulation [26]. Twenty participants per group could provide a statistical power of 90% in the cross-sectional study, and 10 patients in the longitudinal study could provide a statistical power of 80% (Supplementary Table S1). Considering possible drop-out, a total of 22 patients with catathrenia and 22 age-matched non-snoring volunteers were included. All the included participants underwent video/audio PSG at the Sleep Division of Peking University People’s Hospital. The inclusion criteria for the catathrenia group were as follows: 1) age between 18 and 40 y; 2) catathrenia confirmed by overnight PSG and not combined with other sleeping disorders; and 3) informed consent to participate in the study.

The control group consisted of age-matched non-snoring volunteers with the catathrenia group. The inclusion criteria included: 1) no self-report or previously diagnosed sleep problems, including snoring, insomnia, or poor sleep quality; 2) no sleeping disorders confirmed by overnight PSG; and 3) informed consent to participate in the study.

The exclusion criteria for participants in both groups included: 1) taking antibiotics and anti-inflammatory drugs in the past 3 months; 2) having systemic diseases, autoimmune diseases, and congenital diseases; 3) having a history of infection within the past 3 months; 4) missing teeth except for third molars, unfilled caries, probing depth of 11, 31, 16, 26, 36, 46 >4 mm, bleeding on probing >3 (bleeding along gingival sulcus) [27], oral mucosal disease, undergoing dental treatment, and regular use of mouthwash; 5) specific eating habits, such as vegetarian; 6) having pets at home; and 7) unable to collect ≥3 mL saliva samples within 10 mins.

## Polysomnography

Full-night video/audio PSG was recorded using Compumedics E-series (Compumedics, Abbotsford, Victoria, Australia). The details of the PSG recording were described elsewhere [28]. All the records were manually scored by certified technicians and verified by a researcher, who did not participate in the screening of participants and data analysis. Standards from the 2012 American Academy of Sleep Medicine (AASM) manual were used to score sleep architectures, arousals, and respiratory events [29]. The apnea-hypopnea index (AHI) was calculated as apnea and hypopnea events per hour of sleep. Groaning events were defined as deep inspiration followed by a prolonged expiration during which a monotonous vocalization was produced, or an exhalation like a sigh produced at the end of the expiration, without oxygen desaturation. The severity of catathrenia was evaluated by the groaning index (GI, groaning events per hour of sleep).

## Mandibular advancement device (MAD)

All patients with catathrenia included in the study were treated with a custom-fit MAD to stabilize the mandible in a protrusive position during sleep; the design and efficacy of the device have been described in the previous study [8]. Ten patients consented to undertake a post-treatment PSG examination with the MAD after a one-month follow-up, and their saliva samples were collected the following morning after the PSG.

## Saliva samples collection

All the saliva samples were collected according to the microbiome sampling protocol of the Human Microbiome Project [30]. Saliva samples of all participants were collected at 6 to 7 AM, immediately after finishing PSG monitoring, consistent with previous studies [16–18]. Participants were asked to refrain from drinking, eating, or brushing their teeth, to sit in a comfortable position, and to let the saliva drool into a 15 mL collection tube. A minimum of 3 mL unstimulated saliva samples were collected and immediately placed on ice and transferred to the laboratory within 1 h, equalized to 1 ml by pipettes, centrifuged at 10,000 *g* for 10 min at 4°C, with the bacterial pellets stored separately at −80°C until further analyses.

## DNA extraction and 16S rRNA gene sequencing

Total genomic DNA extraction was performed using QIAamp DNA mini kit (Qiagen, Hilden, Germany) according to the manufacturer’s protocol. Briefly, DNA was quantified using a NanoDrop NC2000 spectrophotometer (Thermo Fisher Scientific, Waltham, MA, USA), and quality was checked by agarose gel electrophoresis. Variable regions V3-V4 of the bacterial 16S rRNA gene were amplified with degenerate PCR primers. Sample-specific 7-bp barcodes were incorporated into the primers for multiplex sequencing. The PCR components contained 5 μL buffer (5x), 0.25 μL Fastpfu DNA Polymerase (5 U/μl), 2 μL (2.5 mm) dNTPs, 1 μL (10uM) each forward and reverse primer, 1 μL DNA template, and 14.75 μL ddH_2_O. Thermal cycling included initial denaturation at 98°C for 5 min, 25 cycles of denaturation at 98°C for 30s, annealing at 53°C for 30s, extension at 72°C for 45 s, and a final extension of 5 min at 72°C. The Vazyme VAHTSTM DNA Clean Beads (Vazyme, Nanjing, China) and the Quant-iT PicoGreen dsDNA Assay Kit (Invitrogen, Carlsbad, CA, USA) were utilized for the PCR amplicons purification and quantification. The validated libraries were used for sequencing on an Illumina NovaSeq^TM^ 6000 SP Reagent Kit (500 cycles) using paired-end 2 × 250 bp sequencing at Shanghai Personal Biotechnology Co., Ltd (Shanghai, China). Samples were sequenced in the same run to prevent batch effects.

## Bioinformatics analysis

The sequence data were analyzed using QIIME2 [31], R 4.3.0 (The R Foundation for Statistical Computing, Vienna, Austria), and Python 3.12.2. Raw sequences were denoised and dereplicated with the Divisive Amplicon Denoising Algorithm 2 (DADA2) plugin to generate amplicon sequence variants (ASVs) [32]. Taxonomic information was assigned to ASVs using a Naive Bayes classifier algorithm against the Human Oral Microbiome Database (version 15.22) [33].

The mean number of sequences generated per sample was 102,969, and an average of 67,376 per sample passed quality control and was used for final analysis. The sequences assigned at genus and species levels were 24% and 44% in the catathrenia group, and 23% and 46% in the control group. Alpha (α) diversity was assessed by observed species, Chao1 Index, and Faith’s phylogenetic diversity (Faith’s pd). Beta (β) diversity was analyzed by the Bray-Curtis dissimilarities (abundance weighted distance) and visualized with the principal coordinates analysis (PCoA). Linear discriminant analysis (LDA) effect size (LEfSe) analysis was performed to detect differentially inter-group abundant taxa and visualized it as an LDA score histogram. LDA was used to assess the effect size of each feature, with the cutoff value of the LDA score of 2. The Spearman correlation analysis was performed to explore the effect of clinical variables on the salivary microbiota.

## Machine learning algorithms

The differences in taxa between the catathrenia and control groups were studied to identify the marker bacteria that significantly distinguish each group. This study utilized two different algorithms, including the XGBoost and Random Forest Classifier, to build the model. For the cross-validation, values were multiplied 10-fold, with the training and test sets of 90% and 10%, respectively. The performance of the genera in catathrenia and control classification was measured as the area under the curve (AUC).

## Statistical analysis

All the clinical statistical analyses were performed using R 4.3.0 (The R Foundation for Statistical Computing, Vienna, Austria). All parameters were analyzed using the Shapiro-Wilk normality test. Normally- and skewed-distributed variables were summarized as mean (SD) and median (25th, 75th quartile), respectively. Inter-group comparisons were conducted using the independent *t*-test and Mann-Whitney test. Pearson Chi-square test was used to compare categorical variables. Pre- and post-treatment comparisons were analyzed using paired tests. A *p*-value less than 0.05 was considered statistically significant.

## Results

### Study population

There were 22 patients with catathrenia (12 males/10 females) and 22 age-matched (8 males/14 females) non-snoring control volunteers included in the study. The detailed clinical characteristics are shown in Table 1. Inter-group differences in sex, age, and body mass index (BMI) were non-significant. Patients with catathrenia had significantly higher scores on the Epworth sleepiness scale (ESS) and lower scores on the Montreal Cognitive Assessment (MoCA).

**Table 1.** Clinical characteristics between catathrenia and control group.

Clinical characteristics	Overall *N* = 44^a^	Catathrenia *N* = 22^a^	Control *N* = 22^a^	*p*-value^b^
**Demographic characteristics**				
Sex				0.23
Female	24 (55%)	10 (45%)	14 (64%)	
Male	20 (45%)	12 (55%)	8 (36%)	
Age (y)	28 (26, 32)	28 (25, 31)	30 (26, 39)	0.17
BMI (kg/m^2^)	23.2 (3.3)	24.0 (3.5)	22.5 (3.0)	0.25
ESS	7.8 (3.7)	10.1 (3.8)	5.5 (1.5)	<0.001***
MoCA	28.0 (1.9)	27.0 (2.0)	29.0 (1.0)	<0.001***
**PSG parameters**				
TST (min)	410 (37)	422 (33)	399 (39)	0.014*
Sleep efficiency (%)	92.2 (86.8, 94.9)	92.2 (91.9, 94.9)	89.9 (84.8, 93.9)	0.14
Sleep onset latency (min)	11 (6, 17)	11 (5, 11)	16 (7, 19)	0.12
REM latency (min)	89 (75, 118)	89 (77, 100)	95 (74, 145)	0.38
R (%)	20.0 (4.5)	20.1 (4.2)	20.0 (4.9)	0.86
N1%)	10.1 (5.2, 12.6)	10.1 (5.9, 10.1)	9.5 (5.4, 13.4)	0.96
N2%)	50 (7)	49 (7)	50 (8)	0.40
N3%)	20.3 (6.5)	20.6 (7.2)	20.0 (5.8)	0.90
AHI (events/h)	1.4 (0.5, 3.0)	1.1 (0.5, 3.0)	1.5 (0.8, 3.0)	0.59
3%ODI (events/h)	0.80 (0.18, 2.10)	0.70 (0.10, 1.98)	1.05 (0.30, 2.08)	0.73
ArI (events/h)	10.9 (4.6)	11.8 (4.6)	9.9 (4.5)	0.24
Groaning (N)	/	46 (27, 60)	0	/
Groaning duration (s)	/	17 (14, 20)	0	/
Groaning index (events/h)	/	6.6 (3.7, 8.4)	0	/

^a^n (%); Median (IQR); Mean (SD).

^b^Pearson’s Chi-squared test; Wilcoxon rank sum test.

*p < 0.05; ***p < 0.001.

PSG: polysomnography; TST: total sleep time; REM/R: rapid eye movement sleep; N: non-REM sleep; AHI: apnea-hypopnea index; ODI: oxygen desaturation index; ArI: arousal index.

The PSG parameters between groups were non-significant, indicating well-preserved sleep architectures in catathrenia, except that the control group had statistically shorter total sleep time (TST). Patients with catathrenia had median groaning events of 46 (27,60) episodes (groaning index of 6.6 [3.7, 8.4] events/h), with a median groaning event duration of 17 (14,20) s.

### Comparisons of salivary microbiota characterization

**Figure 2.**
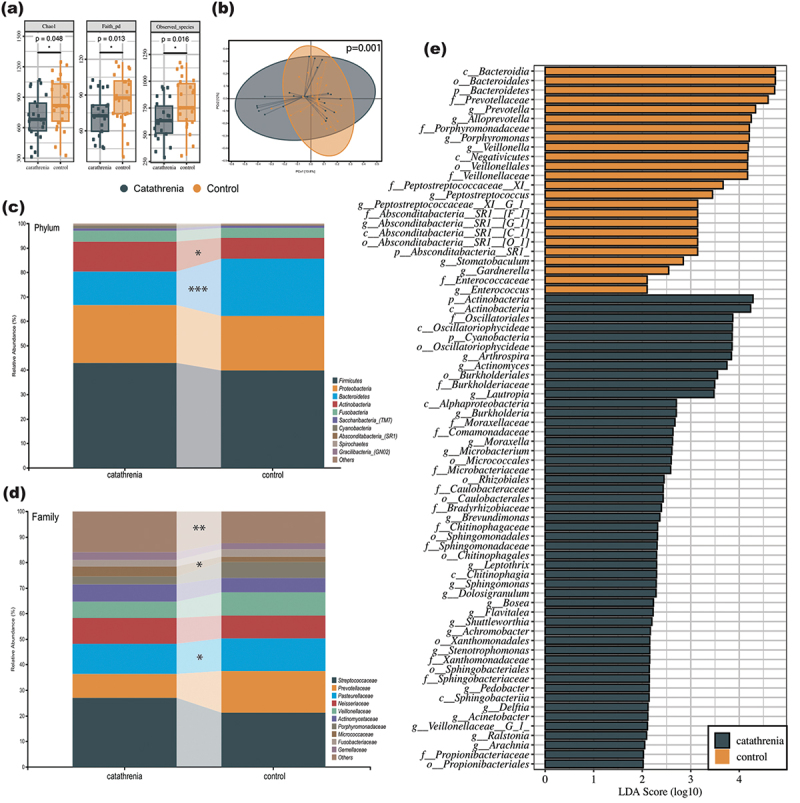
Salivary microbiota distribution of patients with catathrenia and control groups.

The α-diversity of catathrenia, represented by Chao 1, Faith’s pd, and observed species, was significantly lower than that of the control (Figure 2a). Principal coordinate analysis (PCoA) based on Bray-Curtis’s distance revealed inter-group differences in β-diversity, with *p* = 0.001 in the PERMANOVA analysis (Figure 2b).

(a) α-diversity (Chao 1, Faith’s pd, and observed species) of catathrenia and control; (b) β-diversity (principal coordinate analysis, Bray-Curtis’s distance) of catathrenia and control; (c) Composition of the salivary microbiota at the phylum level in catathrenia and control; (d) Composition of the salivary microbiota at the family level in catathrenia and control; (e) The discriminative analysis in catathrenia and control groups according to LEfSe. *p < 0.05; **p < 0.01; ***p < 0.001.

The differences in abundance in taxa were observed, with the relative abundance of *Actinobacteria* statistically increased (*p* = 0.023) and *Bacteroidetes* decreased (*p* = 9.7 × 10^−4^) in catathrenia, compared with that in the control group at the phylum level (Figure 2c). At the family level, *Micrococcaceae* showed higher abundance in patients with catathrenia than in the non-snoring control group (*p* = 0.036). In contrast, *Pasteurellaceae* and *Porphyromonadaceae* showed lower abundance (Figure 2d).

The linear discriminant analysis effect size (LEfSe) analysis with LDA > 2 and *p* < 0.05 as the threshold was conducted (Figure 2e), and there were 137 taxa detected with significant differences, of which 89 and 48 taxa were enriched in catathrenia and control groups, respectively. *Oscillatoriales* and *Burkholderiaceae* were the main representative family-level taxa in catathrenia, and *Prevotellaceae* were representative of the control group. The relative abundance of 21 genera was enriched in patients with catathrenia, and the control group had 10 enriched genera. The three most enriched genera of the two groups are shown in Supplementary Fig. 1, with *Actinomyces*, *Arthrospira*, and *Lautropia* enriched significantly in the catathrenia group.

### Identification of characteristic salivary microbiota in catathrenia

**Figure 3.**
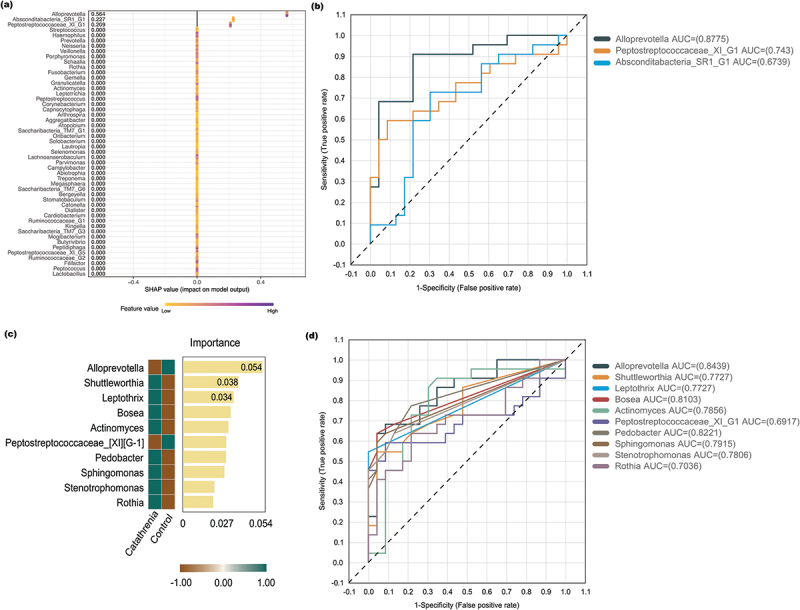
Machine-learning based biomarkers identification and model performance.

Firstly, with the 10-fold cross-validated XGBoost algorithm (Figure 3a), the three most important genera with descending importance were *Alloprevotella* (importance 0.564), *Absconditabacteria_SR1_G1* (importance 0.227), and *Peptostreptococcaceae_XI_G1* (importance 0.209). The AUC of these genera are shown in Figure 3b. Secondly, the nested cross-validated Random Forest Classifier at the genus level was conducted and identified the 10 most important genera that differentiate catathrenia and control groups (Figure 3c). These genera with descending importance were *Alloprevotella* (importance 0.054), *Shuttleworthia* (importance 0.038), *Leptothrix* (importance 0.034), *Bosea*, *Actinomyces*, *Peptostreptococcaceae_XI_G1*, *Pedobacter*, *Sphingomonas*, *Stenotrophomonas*, and *Rothia*. ROC curve was performed and is shown in Figure 3d. The relative abundance of these genera between catathrenia and control is shown in Supplementary Fig. 2.

(a) The most important genera identified by the 10-fold cross-validated XGBoost algorithm. SHAP: SHapley Additive exPlanations. (b) The receiver operating curve (ROC) of top three genera of XGBoost algorithm; (c) The most important genera identified by the nested random forest classifier; (b) The receiver operating curve (ROC) of top 10 genera of the Random Forest Classifier. AUC: area under the ROC curve.

### Effects of MAD on salivary microbiota

**Figure 4.**
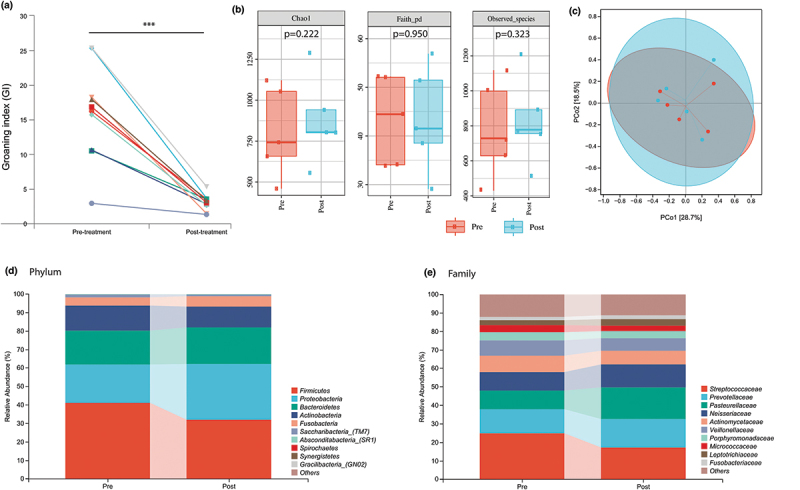
Pre- and post-MAD treatment salivary microbiota distribution in patients with catathrenia.

To evaluate the effects of MAD on salivary microbiota in patients with catathrenia, a one-month longitudinal follow-up study was conducted. Saliva samples were collected following a consistent protocol, and both pre- and post-treatment samples were sequenced in the same batch to minimize batch effects. With MAD, patients’ groaning index (GI) decreased significantly from 15.9 ± 6.8 to 1.1 ± 0.4 events/h (*p* < 0.001, Figure 4a).

MAD: mandibular advancement device. (a) Pre- and post-treatment groaning index changes; (b) Pre- and post-treatment α-diversity (Chao 1, Faith’s pd, and observed species); (c) Pre- and post-treatment β-diversity (principal coordinate analysis, Bray-Curtis’s distance); (d) Pre- and post-treatment composition of the salivary microbiota at the phylum level; (e) Pre- and post-treatment composition of the salivary microbiota at the family level.

The pre- and post-treatment changes of α-diversity, represented by Chao 1, Faith’s pd, and observed species, were insignificant (Figure 4b). Principal coordinate analysis (PCoA) based on Bray-Curtis’s distance revealed non-significant inter-group differences in β-diversity (Figure 4c). At the phylum and family levels, insignificant changes of abundance in taxa were observed (Figures 4d and 4e).

**Figure 5.**
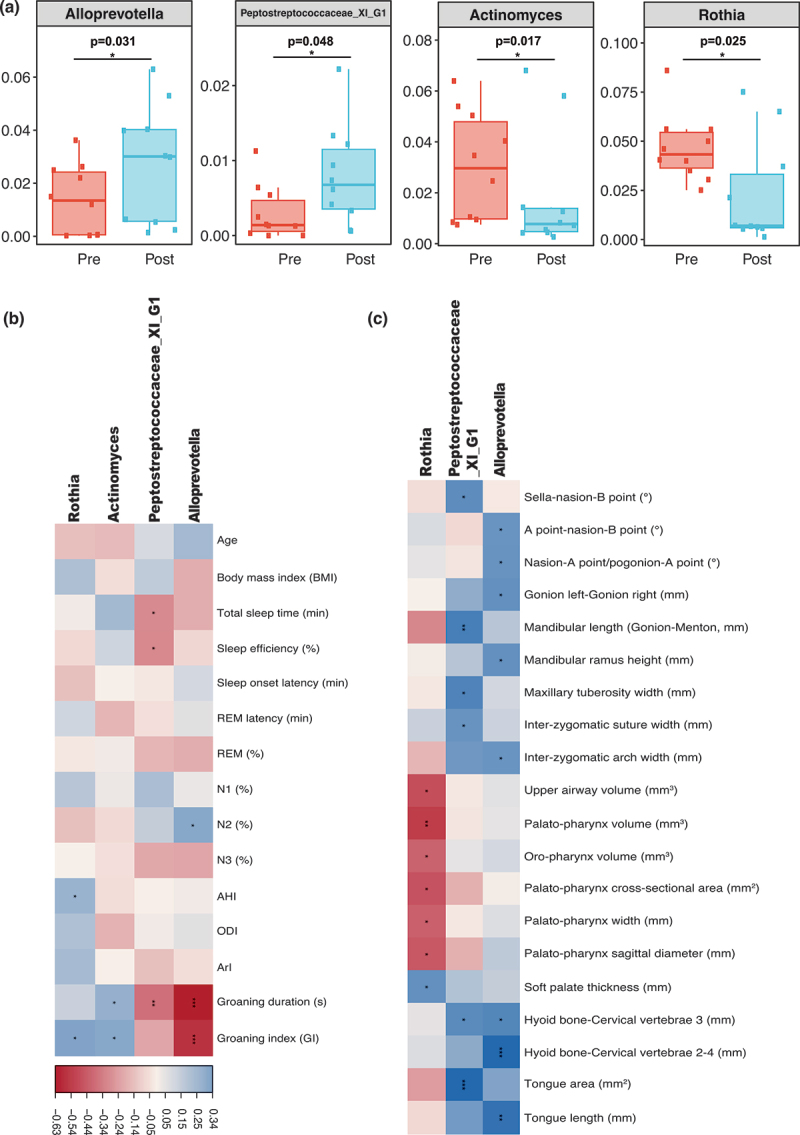
Characteristics of potential biomarker genera in salivary microbiota.

Among the three most important genera identified in the XGBoost algorithm, *Alloprevotella* (*p* = 0.031) and *Peptostreptococcaceae_XI_G1* (*p* = 0.048) showed significant increases following the treatment of MAD. For the top 10 most important genera identified in the random forest algorithm, *Actinomyces* (*p* = 0.017) and *Rothia* (*p* = 0.025) decreased statistically (Figure 5a). The remaining genera identified in the machine learning algorithms did not change significantly.

(a) Pre- and post-treatment abundance of *Alloprevotella*, *Peptostreptococcaceae_XI_G1*, *Actinomyces*, and *Rothia;* (b) Spearman correlation analysis between potential biomarker genera and polysomnographic parameters; REM: rapid-eye movement stage; N: non-REM sleep stage; AHI: apnea-hypopnea index; ODI: oxygen desaturation index; ArI: arousal index. (c) Spearman correlation analysis between potential biomarker genera and craniofacial structures. *p < 0.05; **p < 0.01; ***p < 0.001.

To further explain the potential mechanisms of these genera, spearman correlation analysis was performed with polysomnographic and craniofacial anatomical characteristics (Figures 5b and 5c). The procedures of upper airway cone-beam computed tomography (CBCT) were described elsewhere [34,35], and the explanation of the parameters was detailed in Supplementary Fig. 3. *Alloprevotella* was negatively correlated with groaning event duration (r^2^= −0.63, *p* < 0.001) and groaning index (r^2^= −0.57, *p* < 0.001), and positively correlated with hard tissue structures in the craniofacial area. *Peptostreptococcaceae_XI_G1* was negatively correlated with groaning duration (r^2^= −0.37, *p* = 0.008) and positively correlated with tongue area (r^2^ = 0.82, *p* < 0.001). *Rothia* was negatively correlated with the palato-pharynx volume (r^2^= −0.70, *p* = 0.006) and other upper airway and surrounding soft tissue parameters.

## Discussion

To the best of our knowledge, this is the first study investigating salivary microbiota in patients with catathrenia. A decreased diversity of the oral microbiota and a different distribution of microbiota at the phylum and family levels were identified in the saliva of patients with catathrenia compared with the non-snoring control group. The treatment of MAD did not alter the diversity and distribution of salivary microbiota. Among the most important genera identified in the machine learning algorithms, *Alloprevotella*, *Peptostreptococcaceae_XI_G1*, *Actinomyces* and *Rothia* changed significantly with MAD treatment. These genera were found to be correlated with groaning events and craniofacial structures.

In the present study, patients with catathrenia were found to have decreased α-diversity than the control group, indicating a low richness and diversity of the salivary microbiota. However, high α-diversity is considered a healthy state of the human microbiome [36,37]. Furthermore, the results of β-diversity, represented by the Bray-Curtis’s distance, showed that the salivary microbiota of patients with catathrenia and controls had significant differences and could be distinguished by bacterial composition, suggesting different community evolution structures. The salivary microbiota composition differed significantly between catathrenia and control at the phylum and family levels. However, the α-diversity and β-diversity did not change significantly post-MAD treatment, nor did the distribution of salivary microbiota at the phylum and family levels. Previous findings also demonstrated insignificant changes in microbiota diversity in pediatric patients with OSA after adenotonsillectomy [17], suggesting the treatment of SRBD might affect the abundance of certain genera rather than the composition in salivary microbiota.

In this study, the cross-validated XGBoost and nested Random Forest Classifier algorithms identified the three and ten most important genera as potential biomarkers for distinguishing catathrenia and control, respectively. Among these genera, *Alloprevotella* and *Peptostreptococcaceae_XI_G1* exhibited significant increases post-treatment, while *Actinomyces* and *Rothia* showed statistically significant decreases, indicating that these may serve as potential biomarkers sensitive to treatment for SRBD.

The relative abundance of *Alloprevotella* was significantly lower in catathrenia than in the control group, and it enriched statistically after treatment with MAD. Moreover, this study found the abundance of *Alloprevotella* was significantly correlated with the severity of catathrenia (groaning index and groaning duration). *Alloprevotella*, a gram-negative genus of bacteria from the family of *Prevotellaceae*, has been documented to be related to diseases including autism spectrum disorder, insomnia, and OSA [12,13,19]. *Alloprevotella* is closely associated with the production of short-chain fatty acids (SCFAs), including propionate, butyrate, and acetate [38,39]. In animal models with inflammatory bowel disease (IBD), polygonati odorati rhizoma treatment could enrich gut *Alloprevotella* and SCFAs production. Animal models with atherosclerosis also showed an increase in gut *Alloprevotella* after treatment, and the abundance of *Alloprevotella* negatively correlated with serum lipid profiles [40]. In sleeping disorders, SCFAs have been confirmed to play a role in the modulation of the inflammatory response of gut dysbiosis [41] and cognitive impairment caused by sleep deprivation [14]. However, the association between salivary *Alloprevotella* and catathrenia needs further investigation.

Compared with the control group, patients with catathrenia had significantly lower salivary *Peptostreptococcaceae_XI_G1*, and the abundance increased statistically post-treatment. The peptostreptococcaceae are a family of gram-positive bacteria. Previous studies found that patients with COVID-19 had less abundant *Peptostreptococcaceae_XI_G1* than non-infected participants [42]. *Peptostreptococcaceae_XI_G4* and *Peptostreptococcaceae_XI_G5* were associated with periodontitis [43]. Furthermore, obese individuals had increased *Peptostreptococcaceae_XI_G1* [44], whereas the current study did not yield an association between its abundance and BMI in the correlation analysis. On the other hand, *Peptostreptococcaceae_XI_G1* was correlated with sleep efficiency, groaning duration, and mandibular hard tissue structures. Future studies are needed to clarify the impact of *Peptostreptococcaceae_XI_G1* on catathrenia.

This study found *Actinomyces* to be significantly enriched in patients with catathrenia than in the control group and statistically decreased post-MAD treatment. *Actinomyces* are gram-positive anaerobic to microaerophilic bacteria that typically colonize the human mouth, urogenital tract, and gastrointestinal tract [45]. Previous studies have demonstrated associations between *Actinomyces* and OSA with tonsillar hypertrophy [46], with its relative abundance decreasing after adenotonsillectomy [17], consistent with the current findings. However, Chen et al. found the abundance of *Actinomyces* significantly lower in patients with both OSA and hypertension than in those with OSA [16]. It has been suggested that *Actinomyces* may be associated with mucosal trauma [47], and the enrichment of *Actinomyces* in patients with sleep-related breathing disorders (SRBD) is likely attributable to upper airway obstructions, warranting further investigation.

The relative abundance of *Rothia* was higher in patients with catathrenia than in the control group, but without significant difference; however, it decreased statistically with MAD treatment. Previous studies also found patients with OSA had enriched *Rothia* in salivary microbiota [16], and Wu et al. found that the abundance of *Rothia* was correlated with the severity of OSA (AHI) [48]. The correlation analysis in the current study suggested *Rothia* was significantly associated with upper airway volume and palato-pharynx structures. Yu et al. investigated the craniofacial characteristics of catathrenia, and found patients with catathrenia had narrow hypo-pharynx compared with normal control [35]. Therefore, the enriched *Rothia* could be explained by increases in the upper airway volume following MAD treatment. These findings need to be verified in a causal-effect study design.

Inter-group comparisons using LEfSe analysis, machine learning algorithms, and correlation analysis demonstrated a strong agreement in marker bacteria, with all four characteristic genera associated with groaning events. Furthermore, the position of the hyoid bone and the tongue area were significantly correlated with the abundance of *Alloprevotella* and *Peptostreptococcaceae_XI_G1*, while *Rothia* showed a primary correlation with upper airway dimensions. These findings suggest that upper airway obstructions may play a significant role in the mechanisms of catathrenia, consistent with previous research [8]. On the other hand, while MAD primarily enhances upper airway dimensions and increases tongue area, other treatment modalities for catathrenia may yield different characteristic genera. Currently, there is no consensus on the optimal methods for analyzing biomarker microbiota, and new approaches continue to be proposed. The findings of this study should be validated with larger sample sizes and causal-effect study designs. Validated salivary bacterial markers might be developed as point-of-care diagnostic tools for early catathrenia detection and treatment monitoring in the future, and also shed light on the underlying mechanisms between catathrenia and craniomaxillofacial structures.

The current study has several strengths. First, both patients with catathrenia and the control group underwent PSG as the gold standard diagnosis. Second, two different machine learning algorithms were conducted to identify potential biomarkers in salivary microbiota. Third, the potential biomarkers were verified in the longitudinal study pre- and post-treatment. However, there are also some limitations to the study. First, despite the fact that catathrenia is an uncommon sleep disorder, the sample size of the study is small. The current study did not follow up with participants in the control group to analyze salivary microbiome changes over time. The underlying mechanisms of perturbations found in the study should be examined in the future, in longitudinal studies with larger sample sizes, in-vitro, or in animal studies, with higher resolution molecular analysis. Second, even though the present study considered antibiotics and anti-inflammatory drugs, systemic diseases, eating habits, etc., as exclusion criteria, the salivary microbiota could be altered due to other perspectives not considered in the current study. Third, the study used unstimulated saliva to represent the post-sleep oral microbiome for consistency and comparability with previous studies. However, collecting stimulated saliva samples is significantly faster and more comfortable for the patient.

## Conclusions

This study used high-throughput sequencing and revealed that the salivary microbiota composition was significantly altered in patients with catathrenia than in the control group. Among the potential biomarkers identified by the cross-validated machine learning algorithms, some characteristic genera, including *Alloprevotella*, *Peptostreptococcaceae_XI_G1*, *Actinomyces*, and *Rothia*, could be potential biomarkers sensitive to MAD treatment. Future studies are needed to confirm and determine the mechanisms underlying these findings.

## Supplementary Material

Supplementary material.docx

## Data Availability

The data presented in the study are deposited in the Sequence Read Archive (https://www.ncbi.nlm.nih.gov/) with the accession number PRJNA1097837.

## Registration

The study was registered at the Chinese Clinical Trial Registry (The etiology of catathrenia based on oral and maxillofacial system, No. ChiCTR-COC-17013239, https://www.chictr.org.cn/showproj.html?proj=22286).
