# Supplementary material for: Preliminary analysis of salivary microbiota in catathrenia (nocturnal groaning) using machine learning algorithms
Source: J Oral Microbiol. 2025 Apr 16;17(1):2489613. doi: 10.1080/20002297.2025.2489613 (PMC12004722; doi:10.1080/20002297.2025.2489613)
Supplement: Supplementary material.docx [file ZJOM_A_2489613_SM4681.docx]

**SUPPLEMENTARY MATERIAL TO**

**Preliminary analysis of salivary microbiota in catathrenia (nocturnal groaning)**

**using machine learning algorithms**

Min Yu ^1,2,3^, Yujia Lu ^1,2,3^, Wanxin Zhang ^1,2,3^, Xu Gong ^1,2,3^, Zeliang Hao ^4^, Liyue Xu ^5^, Yongfei Wen ^5^, Xiaosong Dong ^5^, Fang Han ^5, *^, Xuemei Gao ^1,2,3,*^

^1^ Department of Orthodontics, Peking University School and Hospital of Stomatology, No. 22 Zhongguancun South Avenue, Haidian District, Beijing, 100081, P.R. China

^2^ Center for Oral Therapy of Sleep Apnea, Peking University Hospital of Stomatology, No. 22 Zhongguancun South Avenue, Haidian District, Beijing, 100081, P.R. China

^3^ National Center for Stomatology, No. 22 Zhongguancun South Avenue, Haidian District, Beijing, 100081, P.R. China

^4^ Department of Stomatology, Xuanwu Hospital, Capital Medical University, No. 45 Changchun Street, Beijing, 100053, P.R. China

^5^ Sleep Division, Peking University People’s Hospital, No. 11 Xizhimen South Street, Xicheng District, Beijing, 100044, P.R. China

* Corresponding authors:

Fang Han and Xuemei Gao contributed equally to this work.

Fang Han, Professor, Sleep Division, Peking University People’s Hospital, No. 11 Xizhimen South Street, Xicheng District, Beijing, 100044, P.R. China (E-mail: hanfang1@hotmail.com, Telephone: 86-010-88324204)

Xuemei Gao, Professor, Department of Orthodontics, Peking University School and Hospital of Stomatology, No. 22 Zhongguancun South Avenue, Haidian District, Beijing, 100081, P.R. China (E-mail: [xmgao@263.net](mailto:xmgao@263.net), Telephone: 86-010-82195350, Fax: 86-010-82195350; ORCID: 0000-0001-5690-9385)

**Table of contents**

[**Supplementary Table 1** 3](#_Toc192579943)

[**Supplementary Fig. 1** 4](#_Toc192579944)

[**Supplementary Fig. 2** 5](#_Toc192579945)

[**Supplementary Fig. 3** 6](#_Toc192579946)

# **Supplementary Table 1**

| Sample size  in each group | Effect size ω^2^ | |
| --- | --- | --- |
|  | Statistical power of 80% | Statistical power of 90% |
| 5 | 0.0766 | 0.092 |
| 10 | 0.0311 | 0.0411 |
| 20 | 0.0202 | 0.0269 |
| 30 | 0.00907 | 0.013 |
| 40 | 0.00645 | 0.0105 |

Sample size calculation

# **Supplementary Fig. 1**

Relative abundance of top 3 genera in catathrenia and control groups verified by LEfSe analysis.

LefSe: linear discriminant analysis effect size

(a) to (c): top 3 genera with significant enrichments in patients with catathrenia

(d) to (f): top 3 genera with significant enrichments in the non-snoring control group.

# **Supplementary Fig. 2**

Comparisons of relative abundance between catathrenia and control groups.

**p*<0.05; ***p*<0.01; ****p*<0.001.


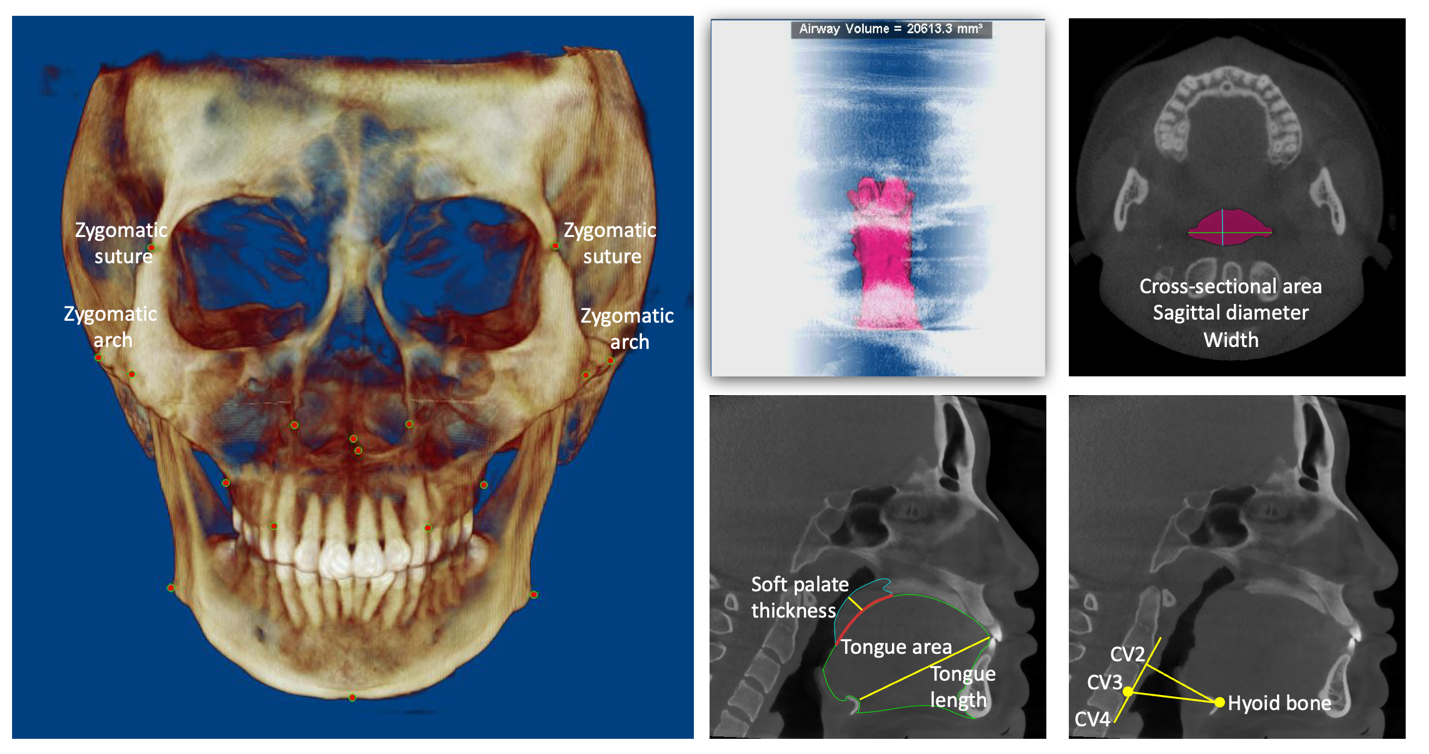

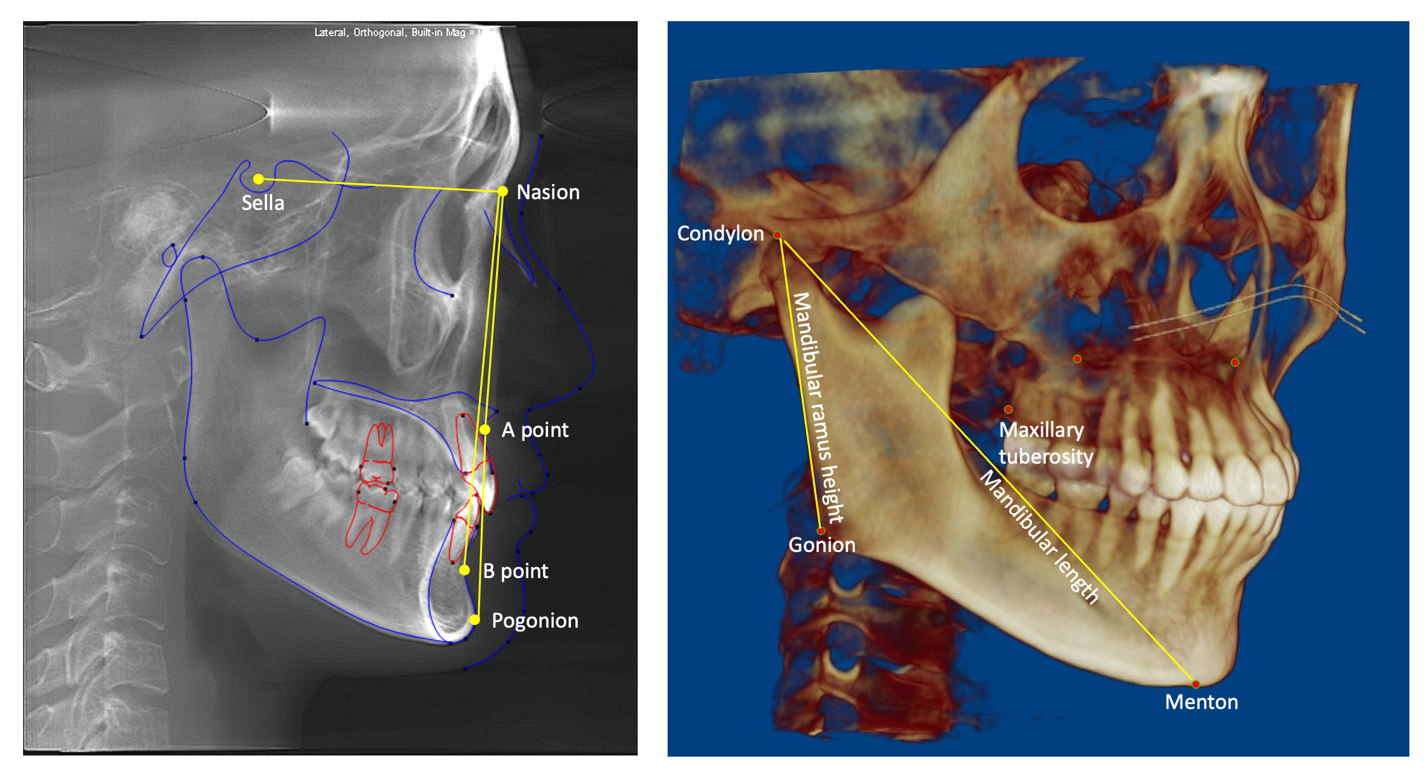
**Supplementary Fig. 3**

Measurement of hard and soft tissue in craniofacial structures.

CV: cervical vertebrae.
